# Sideroflexin 4 affects Fe-S cluster biogenesis, iron metabolism, mitochondrial respiration and heme biosynthetic enzymes

**DOI:** 10.1038/s41598-019-55907-z

**Published:** 2019-12-23

**Authors:** Bibbin T. Paul, Lia Tesfay, C. R. Winkler, Frank M. Torti, Suzy V. Torti

**Affiliations:** 1 Department of Molecular Biology and Biophysics, University of Connecticut Health Center, Farmington, CT 06030 USA; 2 Institute for Critical Technology and Applied Science, Nanoscale Characterization and Fabrication Laboratory, Virginia Tech, Blacksburg, VA 24061 USA; 3 Department of Medicine, University of Connecticut Health Center, Farmington, CT 06030 USA

**Keywords:** Proteins, Mechanisms of disease

## Abstract

Sideroflexin4 (SFXN4) is a member of a family of nuclear-encoded mitochondrial proteins. Rare germline mutations in SFXN4 lead to phenotypic characteristics of mitochondrial disease including impaired mitochondrial respiration and hematopoetic abnormalities. We sought to explore the function of this protein. We show that knockout of SFXN4 has profound effects on Fe-S cluster formation. This in turn diminishes mitochondrial respiratory chain complexes and mitochondrial respiration and causes a shift to glycolytic metabolism. SFXN4 knockdown reduces the stability and activity of cellular Fe-S proteins, affects iron metabolism by influencing the cytosolic aconitase–IRP1 switch, redistributes iron from the cytosol to mitochondria, and impacts heme synthesis by reducing levels of ferrochelatase and inhibiting translation of ALAS2. We conclude that SFXN4 is essential for normal functioning of mitochondria, is necessary for Fe-S cluster biogenesis and iron homeostasis, and plays a critical role in mitochondrial respiration and synthesis of heme.

## Introduction

Mitochondrial diseases are a heterogeneous group of rare genetic disorders^1^. Patients with mitochondrial disease exhibit a range of symptoms and a diverse etiology that in some cases has successfully been traced to specific mutations in mitochondrial proteins. The study of genes contributing to mitochondrial disorders has been an important tool in elucidating basic mechanisms of mitochondrial function as well as in understanding the contribution of mitochondria to cellular and systemic processes.

In 2013 two children with phenotypic evidence of mitochondrial disease and hematopoetic abnormalities were identified^2^. Genomic sequencing identified mutations in sideroflexin 4 (SFXN4), a nuclear-encoded mitochondrial protein in these children. One patient carried a homozygous frameshift mutation resulting in loss of SFXN4 function; the other patient carried a heterozygous mutation that resulted in partial protein activity. Both patients exhibited decreased mitochondrial complex (I-V) activity and red blood cell anisocytosis; macrocytic anemia was present in the more severely affected child. The protein was localized on the mitochondrial inner membrane, and a knockdown experiment in zebrafish demonstrated reduced hemoglobin content and impaired respiration, consistent with a diagnosis of mitochondrial disorder. Apart from this seminal finding, very little is known about the function of SFXN4.

Among the key functions of mitochondria is the synthesis of Fe-S clusters. Mitochondrial Fe-S biogenesis depends on a dedicated multi-protein iron-sulfur cluster (ISC) machinery (see^3–5^). Fe-S cluster formation begins when elemental sulfur is mobilized from cysteine by cysteine desulfurases (NFS1) via a persulfide intermediate for incorporation into nascent Fe-S clusters^4^. Nascent 2Fe-2S clusters are subsequently transferred to apoproteins by a specialized chaperone/co-chaperone system^6^; some clusters undergo further processing to 4Fe4S complexes. In yeast and probably also in higher eukaryotes, initial steps in the biogenesis of cytosolic Fe-S clusters also take place in mitochondria, with subsequent steps occurring in the cytosol^7,8^. Thus, mitochondria are believed to be required for the synthesis of both mitochondrial and cytosolic Fe-S proteins.

Mitochondria also play a key role in heme synthesis. Heme synthesis begins in the mitochondrial matrix with the condensation of succinyl-coenzyme A and glycine by erythoid aminolevulinic acid synthase (ALAS2; specific to erythroid heme synthesis) to form a δ-aminolevulinic acid (ALA). ALA is transported to the cytosol and undergoes a series of enzymatic modifications prior to the final step in heme synthesis, which occurs in mitochondria and is mediated by ferrochelatase (FECH), an Fe-S cluster-containing protein that inserts iron into protoporphyrin IX to form heme^9^. The requirement of FECH for an Fe-S cofactor thus links heme synthesis and Fe-S biogenesis.

In addition to the role of Fe-S clusters in heme synthesis, Fe-S clusters are essential to the function of mitochondrial and cytosolic proteins that participate in numerous cellular processes. Prominent among these is the regulation of iron metabolism. Iron responsive element binding protein 1 (IRP1), one of the two master regulators of cellular iron status, is an Fe-S cluster-containing protein^10^. Under iron replete conditions, the Fe-S cluster in IRP1 remains intact and IRP1 functions as cytosolic aconitase; however, when cellular iron is limited, the Fe-S cluster is disassembled and IRP1 acquires RNA binding activity^11^. In its RNA binding conformation, IRP1 binds to specific iron responsive elements (IREs) present in the 5′ and 3′ untranslated regions (UTR) of target mRNAs such as transferrin receptor (TFR1), ferritin, ferroportin and erythroid-specific ALAS2. IRP2, the second protein that arbitrates levels of intracellular iron, also responds to cellular iron, undergoing degradation by FBXL5, an iron-dependent ubiquitin ligase, only under iron-replete conditions^12,13^. Collectively, these activities of IRP1 and IRP2 act to restore iron levels in situations of iron depletion by increasing synthesis of the iron import protein TFR1 and decreasing levels of proteins that mediate iron storage (ferritin), efflux (ferroportin) and utilization (ALAS2).

In this manuscript, we explore the function of SFXN4 in erythroid cells. We show that SFXN4 is essential for normal functioning of mitochondria, is necessary for Fe-S cluster biogenesis and iron homeostasis, and plays a critical role in mitochondrial respiration and synthesis of heme.

## Results

### SFXN4 knockout dramatically attenuates mitochondrial respiration and causes a shift to glycolysis

**Figure 1:**
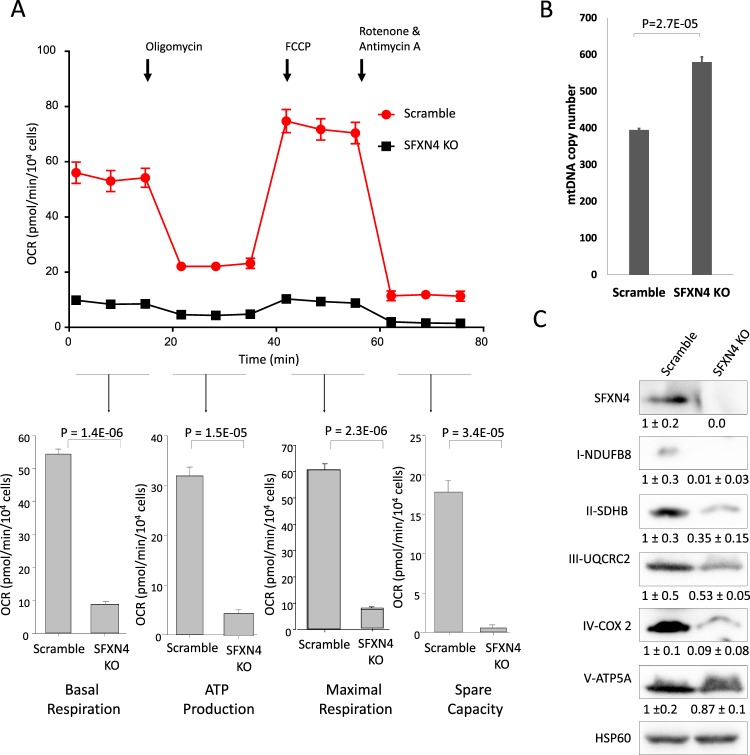
SFXN4 knockout attenuates mitochondrial respiration and affects the steady state of level of respiratory complex proteins. (**A**) Changes in oxygen consumption rate in response to treatment with indicated metabolic inhibitors in K562 (Scramble/SFXN4 KO) cells. Basal respiration, mitochondrial ATP production, maximal respiration and spare capacity were quantified in 8 replicate experiments (means and standard deviation). (**B**) The mean mitochondrial DNA copy number in K562 (Scramble/SFXN4 KO) cells from 3 replicate experiments. (**C**) Immunoblot showing the levels of labile subunits from each of the five mitochondrial respiratory complexes in K562 (Scramble/SFXN4 KO) cells. Means and standard deviations of quantified data from three independent experiments are shown under the blot. Images are representative of three independent experiments.

Since mutational loss of SFXN4 leads to hematopoetic abnormalities, we explored the mechanisms of action of SFXN4 in erythropoietic cells. We used CRISPR/cas9 to knockout (KO) SFXN4 in K562 cells, an erythroleukemic cell line, and tested effects on multiple parameters of respiration using the Agilent Seahorse XF Cell Mito Stress Test assay. SFXN4 knockout resulted in significant decrease in all parameters of respiration, including baseline respiration, respiratory ATP synthesis, maximal respiration, and spare respiratory capacity (Fig. 1A). To confirm that the decrease in respiratory activity did not result from a decrease in the number of mitochondria, we assessed the effect of SFXN4 knockout on mitochondrial DNA copy number. Mean mitochondrial DNA copy number was not decreased, and in fact was modestly increased, by SFXN4 knockout **(**Fig. 1B**)**.

To explore the mechanism by which SFXN4 decreases respiratory activity, we first evaluated the effect of SFXN4 knockout on levels of respiratory complex proteins. Electron transport is carried out by a series of five multiprotein electron transport complexes (I-V). In each of these complexes, we measured levels of a protein that becomes labile when not incorporated into its fully assembled complex. Specifically, we measured the effect of SFXN4 depletion on NDUFB8 (NADH dehydrogenase [ubiquinone] 1 beta subcomplex subunit 8), a protein in complex I; SDHB (succinate dehydrogenase complex iron sulfur subunit B) in complex II; UQCRC2 (ubiquinol-cytochrome c reductase core protein 2) in complex III; COXII (cytochrome c oxidase subunit 2) in complex IV; and ATP5A (ATP synthase F1 subunit alpha) in complex V. Proteins in complexes I-IV were all dramatically decreased in SFXN4 deficient cells; the complex V protein ATP5A was reduced to a more modest extent (Fig. 1C).

**Figure 2:**
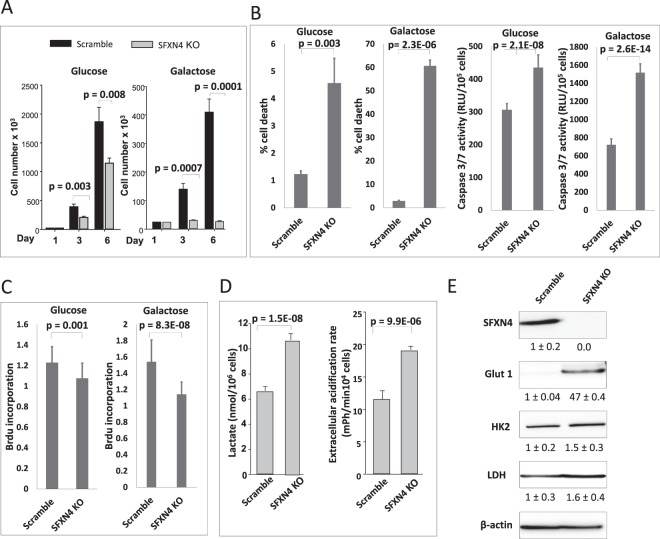
SFXN4 knockout induces lactate secretion and triggers cell death in cells dependent on galactose as a carbon source. (**A**) Cell count measurements of K562 (Scramble/SFXN4 KO) cells cultured in glucose and galactose. (**B**) Percentage of cell death and caspase 3/7 activity in K562 (Scramble/SFXN4 KO) cells cultured in glucose and galactose. (**C**) BrDU incorporation in K562 (Scramble/SFXN4 KO) cells cultured in glucose and galactose. (**D**) Levels of lactate and extracellular acidification rate (ECAR) in the spent medium of K562 (Scramble/SFXN4 KO) cells. Shown are means and standard deviations of 4 replicas in a representative experiment of 3 independent experiments. (**E**) Immunoblot showing the levels of the glucose transporter GLUT1 and indicated glycolytic enzymes in K562 (Scramble/SFXN4 KO) cells.

We next analyzed cell proliferation and viability in a culture medium in which galactose was substituted for glucose. Mammalian cells grown in galactose depend on mitochondrial oxidative phosphorylation for ATP production^14^. In galactose-containing medium, proliferation of SFXN4 knockdown cells was halted completely (Fig. 2A). In contrast, in glucose-containing medium, knockout of SFXN4 exerted only a small effect on proliferation, reducing cell number approximately 40% after 6 days in culture. The reduction in cell number in galactose-containing medium was due to an increase in cell death, as measured by a live/dead dye exclusion assay and by an increase in caspase 3/7 activity (Fig. 2B), as well as a modest but significant decrease in DNA synthesis (Fig. 2C). We tested whether the reduction in mitochondrial respiration was accompanied by a metabolic shift to glycolysis and lactate production. We observed increased levels of extracellular lactate and increased extracellular acidification rate (ECAR) (Fig. 2D) in media from SFXN4 KO cells compared to controls. Consistent with this observation, SFXN4 KO cells dramatically upregulated the expression of glucose transporter GLUT 1 (Fig. 2E). There was no significant change in the levels of hexokinase or lactate dehydrogenase (Fig. 2E). Collectively, these observations suggest that the block in respiration induced by SFXN4 dysfunction leads to a shift from respiratory to glycolytic metabolism.

To test whether depletion of SFXN4 has a similar effect on mitochondrial respiration in non-erythropoietic cells, we used HepG2 (hepatocellular carcinoma) cells. As expected, SFXN4 knockout in HepG2 cells resulted in significant decrease in mitochondrial baseline respiration, respiratory ATP synthesis and spare respiratory capacity compared to the scramble cells (Supplemental Fig. 1A,B). We also observed a decrease in the steady state of respiratory complexes (I-V) in SFXN4 KO HepG2 cells (Supplemental Fig. 1C). In addition, knockout of SFXN4 modestly reduced the ability of HepG2 cells to form colonies (Supplemental Fig. 1D) in normal growth media containing glucose. However, SFXN4 KO HepG2 cells formed almost no colonies when grown in medium in which galactose was substituted for glucose (Supplemental Fig. 1E). Taken together, these results demonstrate that the importance of SFXN4 in mitochondrial respiration extends beyond erythropoetic cells.

### Knockdown of SFXN4 impairs Fe-S cluster biogenesis

**Figure 3:**
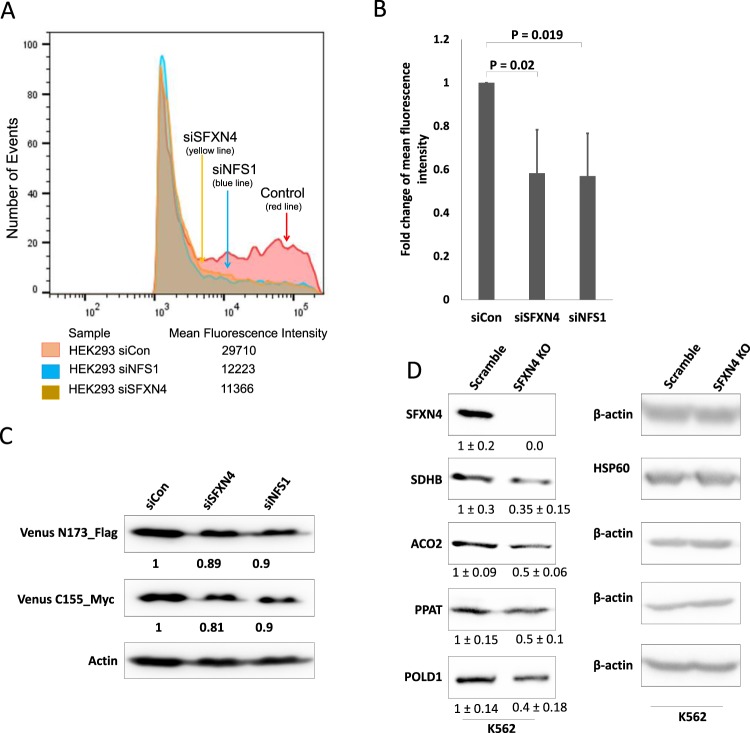
Depletion of SFXN4 reduces Fe-S cluster assembly and affects the stability of Fe-S cluster proteins. (**A**) Relative fluorescence of GRX2-Fe-S dependent sensors in siSFXN4 and siNFS1 transfected HEK293 cells compared to siCon. (**B**) Mean and standard deviation of fold change in fluorescence intensity from three independent experiments. (**C**) Immunoblot showing the relative levels of Venus N173 and C155 (sensor plasmids) in HEK293 cells that were transfected with a siRNA targeted to SFXN4, NFS1, and a control siRNA. (**D**) Immunoblot of indicated Fe-S cluster proteins in SFXN4 KO cells and scramble K562 cells. Images are representative of three independent experiments. Means and standard deviations of quantified data from three independent experiments are shown under the blot. SDHB (succininate dehydrogenase complex iron sulfur subunit **B**), a component of respiratory complex II; ACO2 (mitochondrial aconitase), a citric acid cycle enzyme; PPAT (phosphoribyosyl pyrophosphate amido transferase), an enzyme that catalyzes the first step of the denovo purine nucleotide biosynthetic pathway; POLD1 (large catalytic subunit of DNA polymerase delta), the large catalytic subunit of the DNA polymerase delta (Polδ) complex.

We reasoned that the inhibitory effect of SFXN4 knockout on mitochondrial respiration and stability of multiple respiratory chain complexes might result from a defect in Fe-S cluster biogenesis, since the coordinated delivery of Fe-S clusters to electron transport chain complexes is essential for mitochondrial respiratory function^15^. To test this hypothesis, we used a previously validated Fe-S cluster fluorescence assay (FeSFA) to quantify intracellular Fe-S clusters in living cells^16^. This assay is based on transfection of cells with two plasmids encoding two different fusion proteins: the first encodes the N-terminal half of the Venus fluorescent protein fused to the Fe-S-containing protein glutaredoxin2 (GRX2), and the second encodes the C-terminal half of Venus fluorescent protein fused to GRX2. The fluorescence observed when these two fusion proteins are co-expressed is contingent on GRX2 homodimerization, a process that depends quantitatively on the availability of [2Fe-2S] clusters^16^. Because a high transfection efficiency is required for this assay, we used highly transfectable HEK293 cells. As a positive control, we transfected cells with siRNA to the mitochondrial protein NFS1 (cysteine desulfurase), a key enzyme that supplies the sulfur for Fe-S clusters, and is essential for Fe-S cluster biogenesis^16,17^. Knockdown of both NFS1 and SFXN4 was highly efficient (Supplemental Fig. 2). As shown in Fig. 3A,B, cells with knockdown of either SFXN4 or NFS1 exhibited a strong reduction in mean fluorescence intensity compared with scrambled controls, a direct indication that SFXN4 is involved in Fe-S cluster biogenesis. Controls demonstrated similar levels of expression of Venus fusion proteins in all treatment groups (siCon, siSFXN4 and siNFS1) (Fig. 3C). Neither CIAO1 nor MMS19, two proteins involved in Fe-S cluster assembly and cytosolic targeting of Fe-S clusters (Supplemental Fig. 3A), nor ABCB7, the protein thought to transport Fe-S clusters to the cytosol, were significantly decreased (Supplemental Fig. 3B).

**Figure 4:**
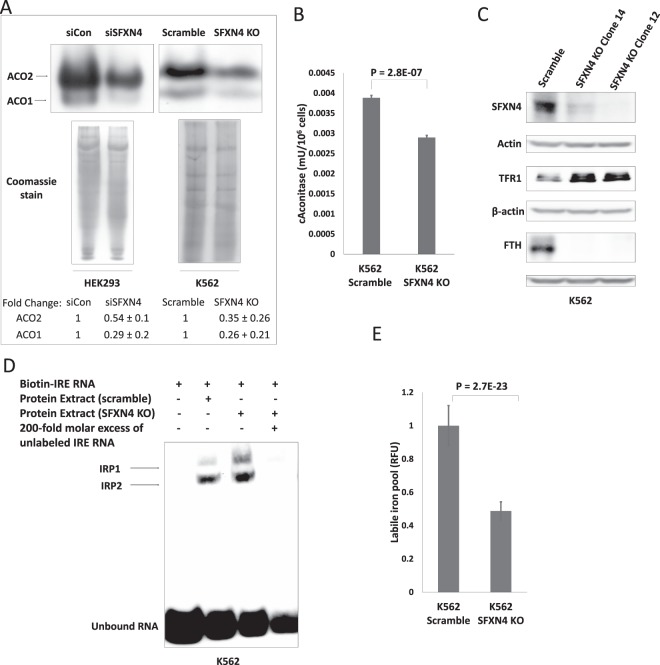
SFXN4 knockout alters cellular iron metabolism. (**A**) In-gel aconitase activity of mitochondrial and cytosolic aconitase. (**B**) Cytosolic aconitase activity in scramble and SFXN4 KO K562 cells. (**C**) Western blot of SFXN4, TFR1 and FTH from scramble and SFXN4 KO K562 cells. (**D**) IRE binding activity of IRP1 and IRP2 in scramble and SFXN4 KO K562 cells determined by gel retardation assay. (**E**) Cytosolic labile iron pool in scramble and SFXN4 KO K562 cells. Shown are means and standard deviations of 4 replicas in a representative experiment of 3 independent experiments. Images are representative of three independent experiments.

Because Fe-S proteins become unstable and are degraded in the absence of an intact Fe-S cluster^18^, we next tested the functional consequences of the reduction in Fe-S cluster formation using western blotting of multiple unrelated Fe-S proteins^19–22^. We examined SDHB^19^ ACO2 (mitochondrial aconitase)^17^, PPAT (phosphoribosyl pyrophosphate amido transferase)^20^ and POLD1 (polymerase delta 1)^18^. These unrelated Fe-S cluster-containing proteins perform diverse functions and in addition are located in different cellular compartments: SDHB and ACO2 are mitochondrial, whereas PPAT and POLD1 are cytosolic, thus allowing us to test the impact of SFXN4 on both mitochondrial and cytosolic Fe-S cluster formation. As shown in Fig. 3D, levels of the mitochondrial Fe-S proteins SDHB and ACO2, as well as the cytosolic Fe-S cluster proteins PPAT and POLD1 were all reduced following SFXN4 knockout. As expected, mRNA levels of these proteins were not decreased by knockout of SFXN4 (Supplemental Fig. 4). Further, not only the levels, but also the activity of enzymes requiring an Fe-S cluster for their function was reduced: as shown in Fig. 4A,B, knockout or knockdown of SFXN4 in K562 and HEK293 cells significantly reduced the activity of both cytosolic and mitochondrial aconitase, both of which require intact Fe-S clusters for catalysis. Levels of representative mitochondrial and cytosolic non-Fe-S-containing proteins were unaffected (Supplemental Fig. 5). Reintroduction of SFXN4 into SFXN4 knockout cells restored protein levels of Fe-S proteins (Supplemental Fig. 6A) and aconitase activity (Supplemental Fig. 6B), supporting the attribution of the effects observed to SFXN4 itself.

### Depletion of SFXN4 exerts complex effects on intracellular iron and mitochondrial morphology

The decrease in cytosolic aconitase activity following knockout of SFXN4 focused our attention on the impact of SFXN4 on overall cellular iron metabolism. When the Fe-S cluster of c-aconitase is disrupted, it not only loses aconitase activity, but simultaneously acquires a new function: the ability to bind to iron regulatory elements (IREs) in the mRNA of proteins that regulate iron transport and storage, such as TFR1 and ferritin. This IRE-binding conformation of c-aconitase is termed IRP1. We tested whether the decrease in c-aconitase activity in SFXN4 knockout cells (Fig. 4A,B) was accompanied by an increase in IRP1 activity. As shown in Fig. 4C, SFXN4 knockout increased levels of TFR1 and reduced levels of ferritin heavy chain (FTH), consistent with activation of IRP1^23^. We then used an electrophoretic mobility shift assay to directly measure the activity of IRP1 following knockdown of SFXN4. This assay indicated a marked increase in IRE binding activity in SFXN4 knockout cells compared to controls (Fig. 4D). We also observed an increase in IRP2 binding activity in SFXN4 knockout cells (Fig. 4D).

To determine whether these effects of SFNX4 knockdown on iron regulatory proteins were sufficient to affect cellular iron, we measured the labile iron pool (LIP), a measure of cytosolic free iron. Despite the increase in IRE binding activity (Fig. 4D) and attendant decrease in ferritin and increase in TFR1 **(**Fig. 4C) (which would be expected to increase labile iron), we observed a decrease in the cytosolic labile iron pool in SFXN4 knockout cells (Fig. 4E).

**Figure 5:**
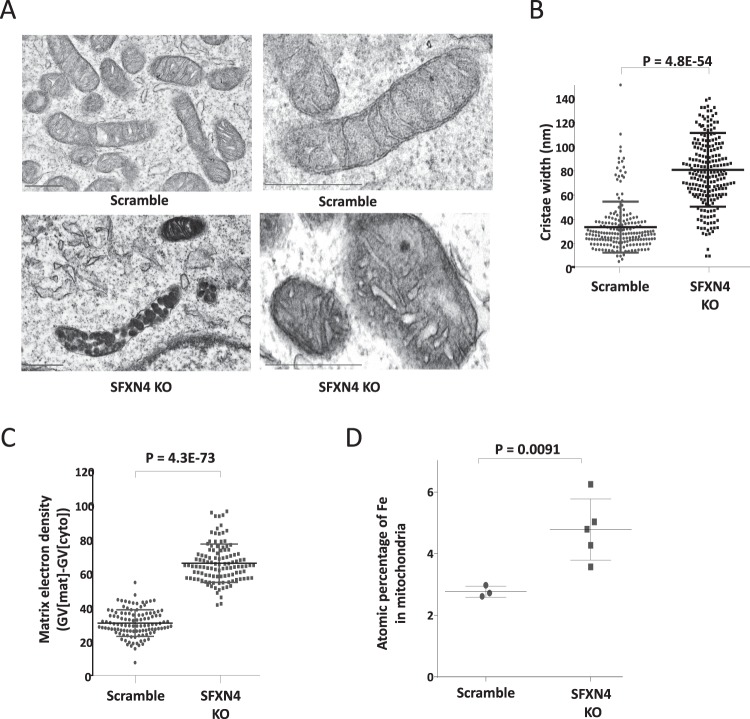
SFXN4 knockout alters mitochondrial morphology and increases mitochondrial iron load. (**A**) Representative transmission electron micrographs of mitochondria from K562 (Scramble/SFXN4 KO)) cells. Scale bars represent 500 nm. (**B**) Measurement of mitochondrial cristae width in K562 (Scramble/SFXN4 KO) cells. (**C**) Quantification of matrix electron density in scramble and SFXN4 KO K562 cells. (**D**) Atomic percentage of Fe in mitochondria of K562 (Scramble/SFXN4 KO) cells determined by TEM-EDS. Mitochondria from 10 or more cells were analyzed to quantify cristae width and matrix electron density.

We reasoned that the observed decrease in cytosolic iron might reflect a redistribution of intracellular iron following SFXN4 knockout, since defects in Fe-S cluster biogenesis frequently result in the repartitioning of intracellular iron such that mitochondrial iron accumulates at the expense of cytosolic iron^24^. To examine whether SFXN4 knockout results in the accumulation of iron in the mitochondria, we first examined mitochondrial ultrastructure using TEM. As shown in Fig. 5A, cristae in mitochondria from SFXN4 knockout cells were rounder and significantly wider than normal (Fig. 5B), a morphological change associated with defective assembly of respiratory chain complexes and lower respiratory efficiency^25^. We also noted a statistically significant increase in electron-dense areas in the mitochondria of SFXN4 KO cells^26^ (Fig. 5C). To determine the identity of this electron-dense material, we performed elemental analysis using spatially resolved EDS (Energy-Dispersive X-ray Spectroscopy). TEM-EDS analysis revealed a statistically significant increase in atomic percentage of iron in mitochondria from SFXN4 KO cells compared to control cells (Fig. 5D). Thus, SFXN4 KO leads simultaneously to cytosolic iron depletion and mitochondrial iron overload.

### SFXN4 depletion impacts key steps in hemoglobinization by decreasing ferrochelatase and promoting IRP1-induced translational inhibition of ALAS2

**Figure 6:**
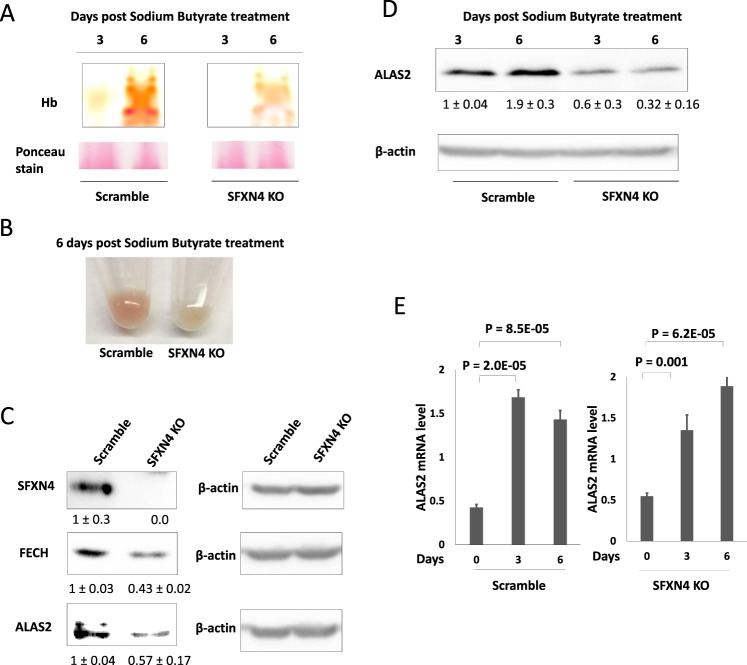
SFXN4 depletion reduces hemoglobinization by destabilizing ferrochelatase (FECH) and by promoting translational inhibition of ALAS2. (**A**) Hemoglobin in differentiated K562 (Scramble/SFXN4 KO) cell at 3 and 6 days post induction of differentiation with sodium butyrate. (**B**) Image of cell pellets 6 days post induction of differentiation with sodium butyrate. (**C**) Western blot of SFXN4, ALAS2 and FECH from K562 (Scramble/SFXN4 KO) cells. (**D**) Western blot of ALAS2 in differentiated K562 (Scramble/CRISPR clone) cells at different time points. Means and standard deviations of quantified data from three independent experiments are shown under the blot. (**E**) Quantification of ALAS2 mRNA in scramble and SFXN4 knockout cells at 0, 3, and 6 days after sodium butyrate. Images are representative of three independent experiments.

Germline mutations in SFXN4 cause anemia and defects in hemoglobinization^2^. To test the effects of SFXN4 knockout on hemoglobin synthesis, we induced erythroid differentiation in K562 SFXN4 knockout cells and scrambled controls using sodium butyrate and measured hemoglobin (Hb) levels using non-denaturing electrophoresis^27^. SFXN4 knockdown substantially reduced hemoglobin levels (Fig. 6A); the decrease was sufficient to result in a visible loss of red color in the cell pellets (Fig. 6B). Reintroduction of SFXN4 into SFXN4 knockout cells restored hemoglobin synthesis (Supplemental Fig. 7A).

Next, we tested whether the inhibitory effects of SFNX4 on hemoglobin synthesis could be traced to its role in Fe-S biogenesis. In particular, we asked whether knockout of SFXN4 affected heme synthesis, since heme plays a regulatory role in hemoglobin synthesis^28^, and ferrochelatase (FECH), the terminal enzyme in heme biosynthesis, requires an Fe-S cluster for maturation and stabilization^21^. FECH protein levels were indeed decreased in SFXN4 knockout cells compared to scrambled controls (Fig. 6C); mRNA levels were not decreased (Supplemental Fig. 7B).

A second mechanism by which a defect in Fe-S biogenesis might affect levels of hemoglobin is by decreasing levels of ALAS2, the erythroid-specific isoform of ALAS that catalyzes the initial and rate-limiting step in heme synthesis^29^. The 5′ untranslated region of ALAS2 mRNA contains an iron-responsive element (IRE)^30^; binding of IRP to this IRE decreases ALAS2 translation. Consistent with the increase in IRP activity following knockout of SFXN4 (Fig. 4), we observed a reduction in protein levels of ALAS2 in SFXN4 KO cells (Fig. 6C) without a decrease in mRNA (Supplemental Fig. 7B). Levels of ALAS2 protein in SFXN4 knockout cells remained reduced following erythropoietic differentiation with sodium butyrate (Fig. 6D), despite the increase in ALAS2 mRNA induced by differentiation (Fig. 6E).

## Discussion

SFXN4 is a member of a family of nuclear-encoded mitochondrial proteins. In metazoans, the sideroflexin protein family comprises five paralogous proteins (SFXN1-5) with variable sequence similarity and partially overlapping tissue expression patterns^31^. Sideroflexin proteins have not been crystalized, but are predicted to have five transmembrane domains and conserved HPDT and asparagine-rich domains of unknown function^31^. SFXN proteins do not possess a canonical mitochondrial targeting sequence^31,32^. Due to the presence of five transmembrane domains, sideroflexin proteins were initially suggested to be a family of mitochondrial carrier proteins with tricarboxylic acid transport activity^33^. However, these findings were subsequently questioned following the discovery of another mitochondrial carrier protein that exclusively transports tricarboxylic acid^31^, as well as the absence of an internal tripartite structure, a defining structural feature of mitochondrial carrier family^34^. Two recent publications shed light on the function of other sideroflexins: SFXN1 was found to be a serine transporter; however, SFXN4 did not compensate for the defects found in sideroflexin 1 mutants^35^. Sideroflexin 2, an outer mitochondrial membrane protein, has 20% sequence homology with SFXN4 and affects the iron content of mitochondria and heme synthesis, but has no effect on iron sulfur cluster formation^36^. Activity of respiratory complex one was also unchanged in a SFXN2 knockout^36^. The function of SFXN4 therefore appears distinct from that of other SFXN family members studied to date.

Here, we identify a previously undescribed role for SFXN4 in iron-sulfur cluster biogenesis. The striking effects of SFXN4 inhibition on Fe-S clusters equaled those of NFS1, a pivotal enzyme that provides sulfur for Fe-S cluster biogenesis (Fig. 3). A role of SFXN4 in Fe-S cluster biogenesis explains the multiple cellular phenotypes we observe following SFXN4 knockout in erythroid cells and is consistent with the clinical picture presented by the children with SFXN4 mutations^2^.

First, we observed that knockout of SFXN4 dramatically reduced respiration and levels of respiratory complexes I-IV (Fig. 1), the four respiratory complexes that contain heme and/or Fe-S complexes^15^. This is reminiscent of other rare disorders of Fe-S cluster biogenesis. For example, patients with ISCU myopathy, who have defects in the scaffold protein that assembles Fe-S clusters, exhibit impaired activity of respiratory complexes, particularly I and II, resulting in an overall reduction in mitochondrial respiration and ATP synthesis^37^. Similarly, depletion of human Ind1, an Fe-S cluster assembly factor for respiratory complex I, decreases complex I activity^19^; and reduction in cellular heme prevents the assembly of complex IV and complex II^38,39^.

The decrease in mitochondrial respiration engendered by knockout of SFXN4 was sufficient to cause a shift to glycolytic metabolism, as evidenced by extracellular acidification, an accumulation of lactate, an increase in GLUT1, and the induction of cell death in cells forced to use respiration for ATP synthesis by substitution of galactose for glucose (Fig. 2). Increased blood lactate is also observed in human patients with SFXN4 deficiency^2^.

The second phenotype of SFXN4 knockout cells explained by an effect of SFXN4 on Fe-S cluster biogenesis is the reduction in levels and activities of functionally unrelated proteins that contain Fe-S clusters (Fig. 3). In the absence of Fe-S clusters, the stability of Fe-S-containing proteins is reduced^20,40^. We observed that SFXN4 knockout depleted levels of Fe-S cluster proteins exhibiting a range of biochemical activities, including mitochondrial aconitase, a hydratase that catalyzes the isomerization of citrate to isocitrate in the citric acid cycle, PPAT, a glycosyl transferase involved in purine biosynthesis, and POLD, a DNA polymerase (Fig. 3). Proteins without an Fe-S cluster were unaffected, indicating that SFXN4 depletion did not non-specifically trigger protein degradation (Supplemental Fig. 5). Further, levels and activities of both mitochondrial and cytosolic proteins were decreased by SFXN4 knockout (Fig. 3), indicating a requirement of SFXN4 for the maturation of both mitochondrial and extra-mitochondrial Fe-S proteins, and suggesting that SFXN4 is involved in a step that precedes exit of activated sulfur complexes from the mitochondria.

The third characteristic of SFXN4 depleted cells that can be traced to a lesion in Fe-S cluster biogenesis is an alteration in iron metabolism, mediated at least in part by the iron regulatory proteins IRP1 and IRP2. Following inhibition of Fe-S cluster formation by SFXN4 knockout, we observed an increase in IRP1 activity, and an attendant decrease in ferritin and increase in TFR1 (Fig. 4). Contributing to these effects on ferritin and TFR1 was an increase in IRP2 (Fig. 4). IRP2 is regulated by FBXL5, an iron-dependent ubiquitin ligase that mediates IRP2 degradation^41^. The increase in IRP2 thus likely ensues from the decrease in labile iron seen in SFXN4 knockout cells (Fig. 4).

Collectively, these changes in IRPs and their targets would be expected to increase intracellular iron. However, we observed a decrease, not an increase, in the labile iron pool (Fig. 4). This apparently paradoxical result is nevertheless in keeping with other diseases of mitochondrial Fe-S cluster biogenesis, where similar decreases in cytosolic iron have been reported. For example, in the mitochondrial diseases Friedreich’s ataxia and ISCU myopathy, defects in Fe-S cluster biogenesis^15,37^ lead to cytosolic iron depletion^24^. Further, similar to observations reported here for SFXN4, a decrease in mitochondrial iron utilization due to defects in Fe-S and heme generation, as well as a compensatory increase in IRP1, have been implicated in the excessive mitochondrial iron accumulation in Freidreich’s ataxia^42–44^. Although the mechanism underlying this redistribution of iron remains uncertain, it has been suggested that failure of Fe-S cluster biogenesis signals an iron-deficient mitochondrial status, leading to increased deposition of iron in mitochondria at the expense of cytosolic iron, thus depleting cytosolic iron^45^.

Supporting this model, studies in both yeast and mammalian systems have shown that defects in proteins involved in Fe-S cluster biogenesis lead to mitochondrial iron overload^26,46^. Similarly, we observed an increase in electron density and a quantitative increase in iron following knockout of SFXN4 (Fig. 5). Thus, both changes in iron metabolism and distribution as well as morphological changes of mitochondria in SFXN4 knockout cells (Fig. 5) are concordant with features observed in other diseases of mitochondrial Fe-S biogenesis.

The fourth phenotype we were able to link to a defect in Fe-S biogenesis are alterations in erythroid cells, important clinical features of patients with mutations in SFXN4, who exhibit mixed macrocytic and microcytic anemia^2^. In particular, we observed a substantial decrease in hemoglobin following SFXN4 knockout in differentiated K562 cells (Fig. 6). We traced this to both a reduction in ferrochelatase, an Fe-S cluster-containing enzyme^47^ that inserts Fe into protoporphyrin IX to form heme **(**Fig. 6**)**, and an IRP-mediated decrease in the erythroid-specific isoform of δ-aminolevulinate synthase (ALAS2), which catalyzes the rate-limiting step in heme synthesis (Fig. 6). Similar to these findings, zebrafish deficient in GRX5, a component of the mitochondrial Fe-S cluster (ISC) machinery^30^, also develop an anemia attributable to IRP1-driven translational inhibition of ALAS2^30^.

Overall, our study demonstrates that SFXN4 is a pivotal protein essential for Fe-S cluster biogenesis, mitochondrial respiration, and the function of Fe-S cluster proteins involved in cellular iron homeostasis and the synthesis of hemoglobin in erythroid cells. Decreases in SFXN4 disrupt key iron-containing respiratory chain complexes, cause a shift to glycolytic metabolism, inhibit key iron regulatory proteins in both the cytosol and mitochondria, and cause mitochondrial iron overload.

## Methods

### Cell culture

K562, HepG2 and HEK293 cells were purchased from American Type Culture Collection (ATCC). K562 and HepG2 cells were grown in RPMI (Gibco) and HEK293 cells were grown in DMEM (Gibco) basal media; both media were supplemented with 10% fetal bovine serum (FBS) (BenchMark™ FBS - Gemini Bio Products) and 100 U/ml penicillin/streptomycin (Invitrogen).

### Gene silencing by siRNA and CRISPR

Transient siRNA-mediated knockdown was performed with ON-TARGETplus siRNAs (humanSFXN4: L-018237-02-0005; NFS1: L-011564-01-0005; and Non-Targeting siGENOME siRNA Pool #2) obtained from Dharmacon. Cells were transfected with 12 nM siRNA using DharmaFECT 1 Transfection Reagent. Transfections were performed twice at 3 day intervals and cells were collected 72 hours after the second transfection. For clustered regularly interspaced short palindromic repeats (CRISPR) knock out, guideRNAs were designed to specifically cut in the first exon of the human SFXN4 gene (Ensemble sequence ENSG00000183605). Guide RNA sequence for SFXN4 was GTGATCCAGAAGCGCACGTT and for scrambled control was CAGTCGGGCGTCATCATGAT. Oligos were purchased from Integrated DNA Technologies Inc. (IDT), phosphorylated, and annealed. The annealed gRNA oligos were cloned into the lentiCRISPRv2 plasmid. Lentivirus was made using the packaging plasmids psPAX2 and pMD2.G (Addgene plasmid #12260 and Addgene plasmid #12259) transfected into HEK293T cells using Lipofectamine 2000 (Thermo Fisher). Forty-eight hours after lentivirus infection, puromycin was added to the culture to select for lentiCRISPR integration. After selection, cells were tested using a T7E1 assay (NEB) to verify the creation of insertions or deletions (INDELs). Once INDELs were verified, cells were plated sparsely to isolate single colonies. The region of CRISPR binding was amplified by PCR and sequenced by Sanger sequencing. Sequencing was performed using TIDE analysis using chromatograms of the edited samples and wildtype cells^48^. Clones that had frameshift mutations in both alleles were expanded, and knockout of SFXN4 confirmed by western blot.

### Transient overexpression of SFXN4

Human SFXN4 cDNA obtained from GE Healthcare Dharmacon Inc. (MGC Human SFXN4 Sequence-Verified cDNA (CloneId:4996745) was sub cloned into pcDNA™3.1 (Invitrogen). K562 cells were transfected with empty and SFXN4 plasmid using Lipofectamine™ LTX Reagent (Invitrogen). Briefly, 5 × 10^5^ K562 cells were plated on a 6 well plate and transfected with 2.5 μg of plasmid DNA. Cells were used for experiments 72 hours post-transfection.

### Western blotting

Western blotting was performed as previously described^49^. Cell lysates were prepared in 1X RIPA buffer (Millipore) supplemented with 1X protease inhibitor cocktail (Roche Diagnostics). Total protein concentration was determined by BCA reagent (Thermo Scientific). Equal amounts of proteins were separated by SDS–polyacrylamide gel electrophoresis and transferred to nitrocellulose membranes. The membranes were then incubated with primary and secondary antibodies respectively. Signals were detected using chemiluminescence substrate (Thermo Scientific). Electron transport proteins were detected using a total OXPHOS human western blot antibody cocktail (ab110411) from Abcam. The primary antibodies used are HSP60 (Cell Signaling Technology (12165 S)), SFXN4 (Thermofischer (PA5-35980)) ACO2 (Cell Signaling Technology (6571S)), PPAT (ProteinTech (15401-1-AP)), POLD1 (BD Biosciences (610972)), NFS1 (Santa Cruz Biotechnology (sc-365308)), AFG3L2 (Proteintech (14631-1-AP)), CPOX (Santa Cruz Biotechnology (sc-393388)), ALAS1 (Abnova (H00000211-Q01)), HSP70 (Cell Signaling Technology (4876S), FTH^49^, TFR1 (Thermo Fisher (13-6800)), FECH (Santa Cruz Biotechnology (sc-377377), Flag (SigmaAldrich (F3165-1MG)), c-Myc (Cell Signaling Technology (2276S)), ALAS2 (Abnova (H00000212-M01), abcam (ab184964)), GLUT1 (Proteintech (21829-1-AP)), Hexokinase II (Cell Signaling Technology (2106S)), LDHA (Proteintech (19987-1-AP)), CIAO1 (Cell Signaling Technology (D1B4G) 81376)), MMS19 AB (Proteintech (16015-1-AP)) and Actin (Sigma Aldrich (A3854).

### Quantitative RTPCR

Total RNA was isolated using High Pure RNA Isolation Kit (Roche) and qRTPCR performed as described^50^. Primer sequences are provided in Supplemental Table 1.

### Seahorse extracellular flux analysis

Oxygen consumption rate and extracellular acidification rate were measured using a Seahorse XF 96 Analyzer (Agilent) with an Agilent Seahorse XF Cell Mito Stress Test Kit. K562 cells, normally grow in suspension, were grown in polylysine-coated culture plates to adhere. Briefly, the day before the analysis, 25,000 cells/well were plated in growth medium and incubated at 37 °C and 5% CO_2_ overnight. On the day of analysis, growth medium was replaced with XF-Base medium containing L-glutamine (2 mM), sodium pyruvate (5 mM) and glucose (10 mM).

### Measurement of secreted lactate

Extracellular lactate was measured by colorimetric L-Lactate assay kit according to the manufactures instruction (Abcam, Inc., Cambridge, MA).

### Mitochondrial DNA copy number

Mitochondrial DNA copy number was measured using Human Mitochondrial DNA (mtDNA) monitoring Primer Set (TaKaRa) according to the manufacturer’s instructions.

### Measurement of c-aconitase and IRE-IRP binding assay

Cytosolic aconitase activity was measured using an aconitase assay kit as per manufacturer’s instructions (Abcam, Inc., Cambridge, MA). RNA binding activity of IRP proteins was determined by RNA electrophoretic mobility shift assay using LightShift™ Chemiluminescent RNA EMSA Kit (Thermo Scientific).

### Fe-S cluster fluorescent assay

Fe-S cluster Fluorescent Assay was performed as described^16^. Fe-S fluorescent sensor plasmids were a generous gift of Dr. Jonathan J Silberg, Department of Biochemistry and Cell biology, Rice University, USA. Prior to transfection with sensor plasmids, siRNA was used to deplete SFXN4 or NFS1. Next, N- and C-terminal Venus-GRX2 fusion constructs were mixed in 1:1 v/v ratio and co-transfected into HEK293 cells using FugeneHD (Promega). After 24 hours, fluorescence was quantified by flow cytometry.

### Cell proliferation, cell death/viability and caspase 3/7 assay

Cell proliferation was measured using the BrdU incorporation assay (Cell Signaling Technology). Viability was determined by flow cytometry using a LIVE/DEAD™ Viability/Cytotoxicity Kit (Thermofisher Scientific). Caspase 3/7 activity was measured using Caspase-Glo® 3/7 Assay Systems (Promega).

### In-gel aconitase activity

In gel aconitase activity was performed as described^51^. Cells were lysed with 1% Triton/citrate Lysis buffer (pH 7.5) (40 mM KCl, 25 mM Tris-Cl, 1% Triton, 1 mM DTT, 2 mM Sodium Citrate and 0.6 mM MnCl2). Protein concentration was estimated with Bradford reagent (Biorad). Samples were loaded onto aconitase activity gels consisting of an 8% separating gel (acrylamide (8%), 132 mM Tris base, 132 mM borate and 3.6 mM citrate) and a 4% stacking gel (acrylamide (4%), 67 mM Tris base, 67 mM borate, 3.6 mM citrate). Running buffer was 25 mM Tris pH 8.3, 192 mM glycine and 3.6 mM citrate. Samples contained 40 µg of protein, 25 mM Tris-Cl (pH 8.0), glycerol (10%) and bromophenol blue (0.025%). Aconitase activity was determined by incubating the gel in the dark at 37 °C in 100 mM Tris (pH 8.0), 1 mM NADP, 2.5 mM cis-aconitic acid, 5 mM MgCl_2_, 1.2 mM MTT, 0.3 mM phenazine methosulfate, and isocitrate dehydrogenase (5U/ml).

### Hemoglobin assay

K562 cells were differentiated using sodium butyrate (1 mM, Sigma-Aldrich) for 3–6 days^52^. Hemoglobin was assessed by native polyacrylamide gel electrophoresis followed by chromophore-enhanced visualization, as described^21^. Cells were lysed in 1X TBS (pH 7.5) (Bio Rad) by sonication and centrifuged for 15 minutes at 4 °C at 20,000 g. Supernatants were collected and total protein concentration was determined. Equal amounts of protein, mixed 1:1 with native gel sample buffer (Invitogen) were subjected to electrophoresis at 125 V. A 4% to 20% Tris-glycine gel with 1X native gel running buffer (Invitogen) was used. Proteins were transferred to nitrocellulose for 1 hour in alcohol-free transfer buffer (Tris base (25 mM) and glycine (192 mM)) using a Bio Rad mini transfer unit. Hemoglobin was visualized by incubating the membrane in buffer containing 50 mM Tris (pH7.4), 50 mM imidazole, 0.5 mg/mL diaminobenzidine, and 0.1% H2O2.

### Labile iron pool (LIP) assay

Labile iron was measured as previously described^53^, 25,000–50,000 cells were seeded in 96-well plates (Black with Clear Bottom purchased from Coring). Cells were loaded with 2 μM calcein acetoxymethyl ester for 15 to 30 minutes at 37 °C, and then washed with PBS. 100 μM starch-conjugated desferrioxamine (DFO; a generous gift of Biomedical Frontiers, Inc., Minneapolis, MN) was added to remove extracellular iron. Fluorescence was measured at 485 nm excitation and 535 nm emission with a fluorescence plate reader (BioTek Synergy 2). After the fluorescence signal was stabilized, isonicotinoyl salicylaldehyde hydrazine (SIH) was added at a final concentration of 10 µM to remove iron from calcein, causing dequenching. The change in fluorescence following the addition of SIH (ΔF) was used as an indirect measure of the labile iron pool. Experiments were performed in triplicate with 8 replicates/point; data are presented as mean values with standard deviations.

### Electron microscopy

Cells were fixed in 2.5% glutaraldehyde in cacodylate (0.1 M) buffer, and post-fixed with 1% osmium tetroxide/0.8% ferricyanide/cacodylate buffer (0.1 M). Following dehydration and embedding in epoxy resin, ultrathin (70 nm) sections were prepared, stained with uranyl acetate and Sato’s lead citrate, and examined with Hitachi H-7650 TEM. Energy-Dispersive X-ray Spectroscopy (EDS) was performed on a JEOL 2100 TEM equipped with a high resolution pole piece and JEOL EDS detector.

### Quantification of electron density and cristae width in TEM micrographs

To quantify mitochondrial matrix electron density, cytosolic gray values were subtracted from mitochondrial matrix gray values using ImageJ software (NIH). Quantification of cristae width was performed by measuring cross sectional width of individual crista using ImageJ software (NIH). 10 or more cells were analyzed.

### Statistics

Experiments were performed at least in triplicate (biological replicates) with 3–8 technical replicates per point. Significance was assessed using Students two-tailed t test, with p ≤ 0.05 accepted as significant. Actual p values for each experiment are shown in the figures.

### Data sharing statement

For original data please contact the corresponding author.

## Supplementary information

Supplementary Information
