# Supplementary material for: Sideroflexin 4 affects Fe-S cluster biogenesis, iron metabolism, mitochondrial respiration and heme biosynthetic enzymes
Source: Sci Rep. 2019 Dec 23;9:19634. doi: 10.1038/s41598-019-55907-z (PMC6928202; doi:10.1038/s41598-019-55907-z)
Supplement: Supplementary file 1 — Supplementary Information [file 41598_2019_55907_MOESM1_ESM.pdf]

Sideroflexin 4 affects Fe-S cluster biogenesis, iron metabolism, mitochondrial respiration and heme biosynthetic enzymes.

Bibbin T. Paul[1], Lia Tesfay[1], C.R. Winkler[2], Frank M. Torti[3], Suzy V. Torti[1]

[1]Department of Molecular Biology and Biophysics, and [3]Department of Medicine, University of Connecticut Health Center, Farmington CT 06030; [2]Institute for Critical Technology and Applied Science, Nanoscale Characterization and Fabrication Laboratory, Virginia Tech, Blacksburg, VA, 24061, U.S.A

Corresponding author:

Suzy V. Torti
Department of Molecular Biology and Biophysics
University of Connecticut Health Center
Farmington CT 06030
Email: storti@uchc.edu
Phone: 860-679-6503
FAX: 860-679-3408

Target Gene		Target Sequence (5'-3')
FECH	Forward	TGG CAC CAT TCA TCG CCA AA
	Reverse	GAT GGA CGT ACC GAA ATC CAA T
SDHB	Forward	GAC ACC AAC CTC AAT AAG GTC TC
	Reverse	GGC TCA ATG GAT TTG TAC TGT GC
ALAS2	Forward	TGT CCG TCT GGT GTA GTA ATG A
	Reverse	GCT CAA GCT CCA CAT GAA ACT
POLD1	Forward	CAG TGC CAA GGT GGT GTA TGG
	Reverse	CTT GCT GAT AAG CAG GTA TGG G
PPAT	Forward	AGC ACC CAC AGC ATA CTC C
	Reverse	ACA CTG GAA TAA GAC GAC CAA TG
ACO2	Forward	CCC TACAGC CTACTG GTG ACT
	Reverse	TGT ACT CGT TGG GCTCAA AGT

Table 1: List of qPCR primers used.

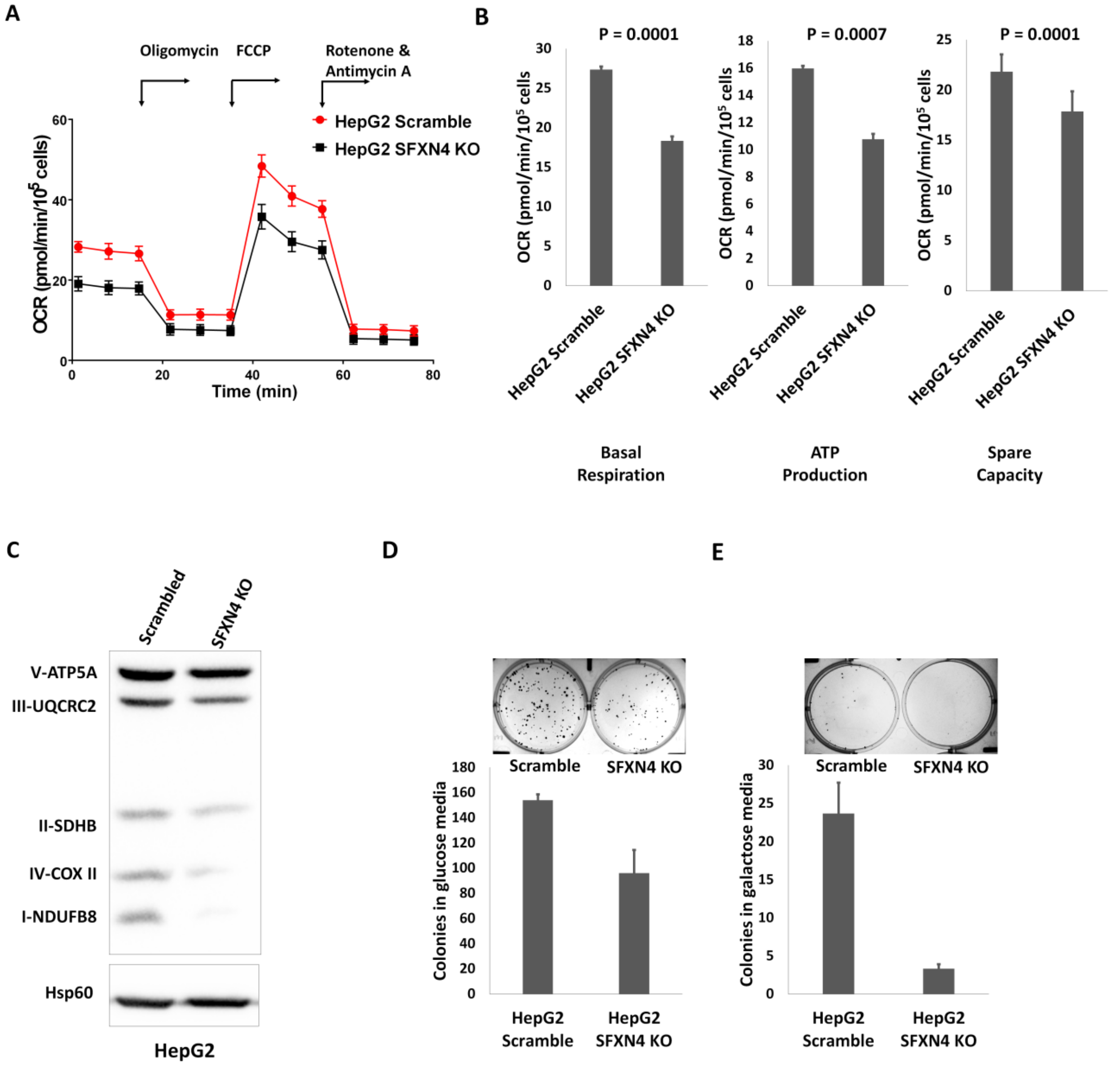

Supplementary Figure 1: SFXN4 knockout attenuates mitochondrial respiration and affects the steady state of level of respiratory complex proteins in HepG2 cells. A) Changes in oxygen consumption rate in response to treatment with indicated metabolic inhibitors in HepG2 (Scramble/SFXN4 KO) cells. B) Basal respiration, mitochondrial ATP production and spare capacity were quantified in 8 replicate experiments (means and standard deviation). C) Immunoblot showing the levels of labile subunits from each of the five mitochondrial respiratory complexes in HepG2 (Scramble/SFXN4 KO) cells. D) Colony formation of HepG2 (Scramble/SFXN4 KO) cells in growth media with glucose. E) Colony formation of HepG2 (Scramble/SFXN4 KO) cells in growth media with galactose substituted for glucose. Images are representative of three independent experiments.

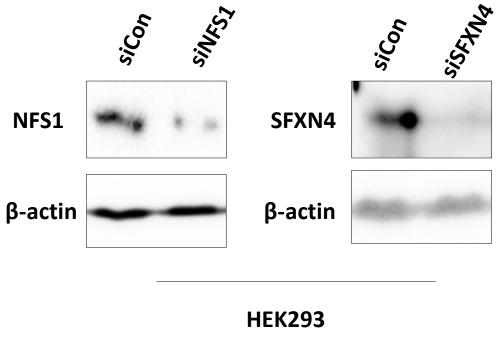

Supplementary Figure 2: Efficient knockdown of SFXN4 and NFS1 in HEK293 cells. Western blot of indicated proteins in HEK293 (siControl/siSFXN4) cells.

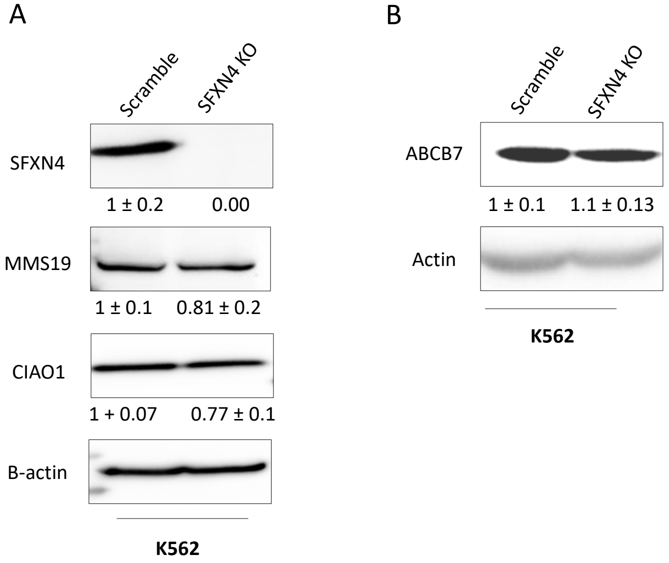

Supplementary Figure 3: SFXN4 knockout does not substantially alter the protein levels of CIA targeting complex proteins. A) Western blot of MMS19 and CIAO1, components of a multiprotein targeting complex that mediates the incorporation of iron-sulfur cluster into various cytosolic and nuclear apoproteins. Differences were not statistically significant, $p>0.05$. B) Western blot of ABCB7, the protein thought to transport Fe-S clusters to the cytosol.

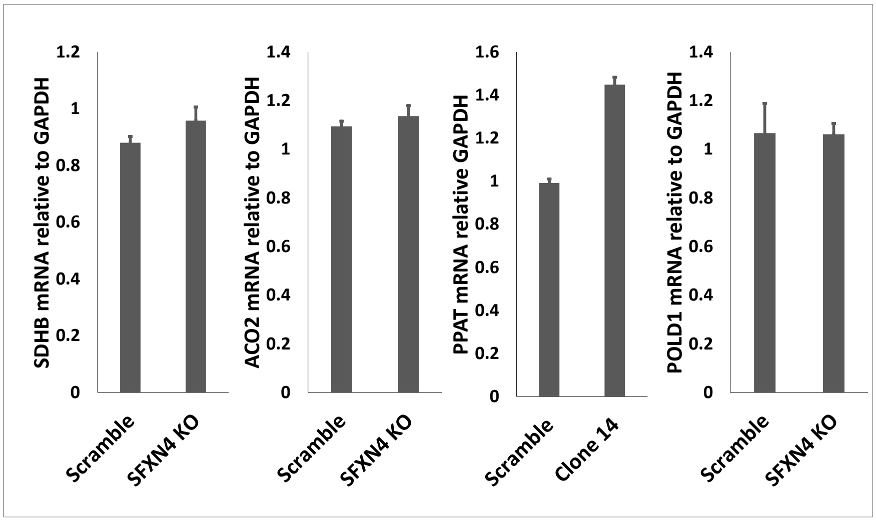

Supplementary Figure 4: SFXN4 knockout does not decrease mRNA levels of Fe-S proteins. mRNA levels of SDHB, ACO2, PPAT and POLD1 in K562 (Scramble/ SFXN4 KO) cells as measured by qRT-PCR.

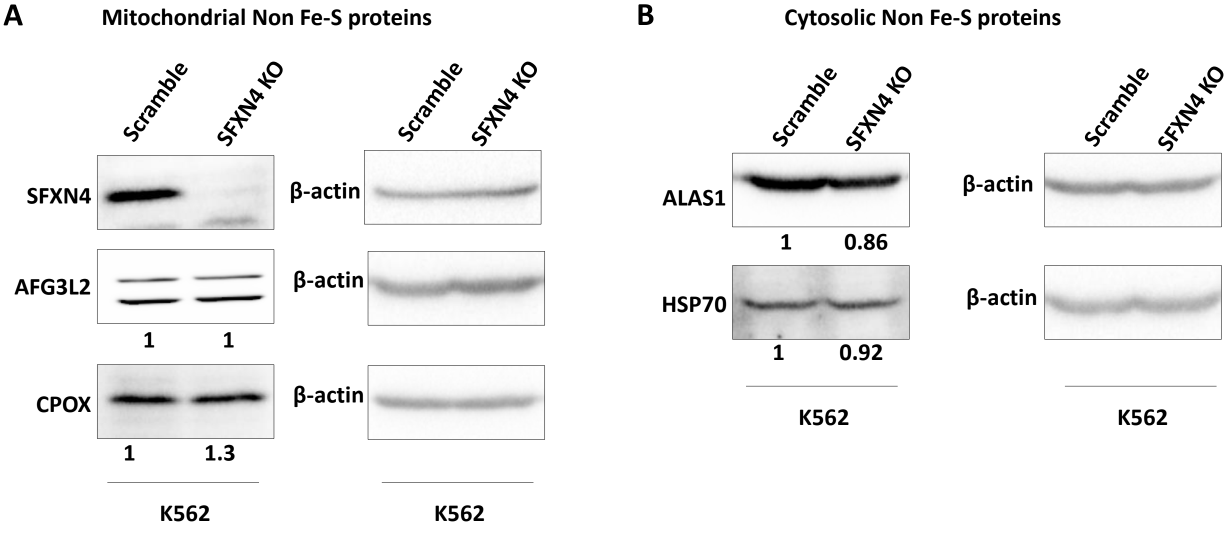

Supplementary Figure 5: SFXN4 knockout does not change levels of mitochondrial or cytosolic non Fe-S containing proteins. The mitochondrial proteins AFG3L2 and CPOX and the cytosolic proteins ALAS1 and HSP70 were measured in K562 scramble and SFXN4 knockout cells by western blotting.

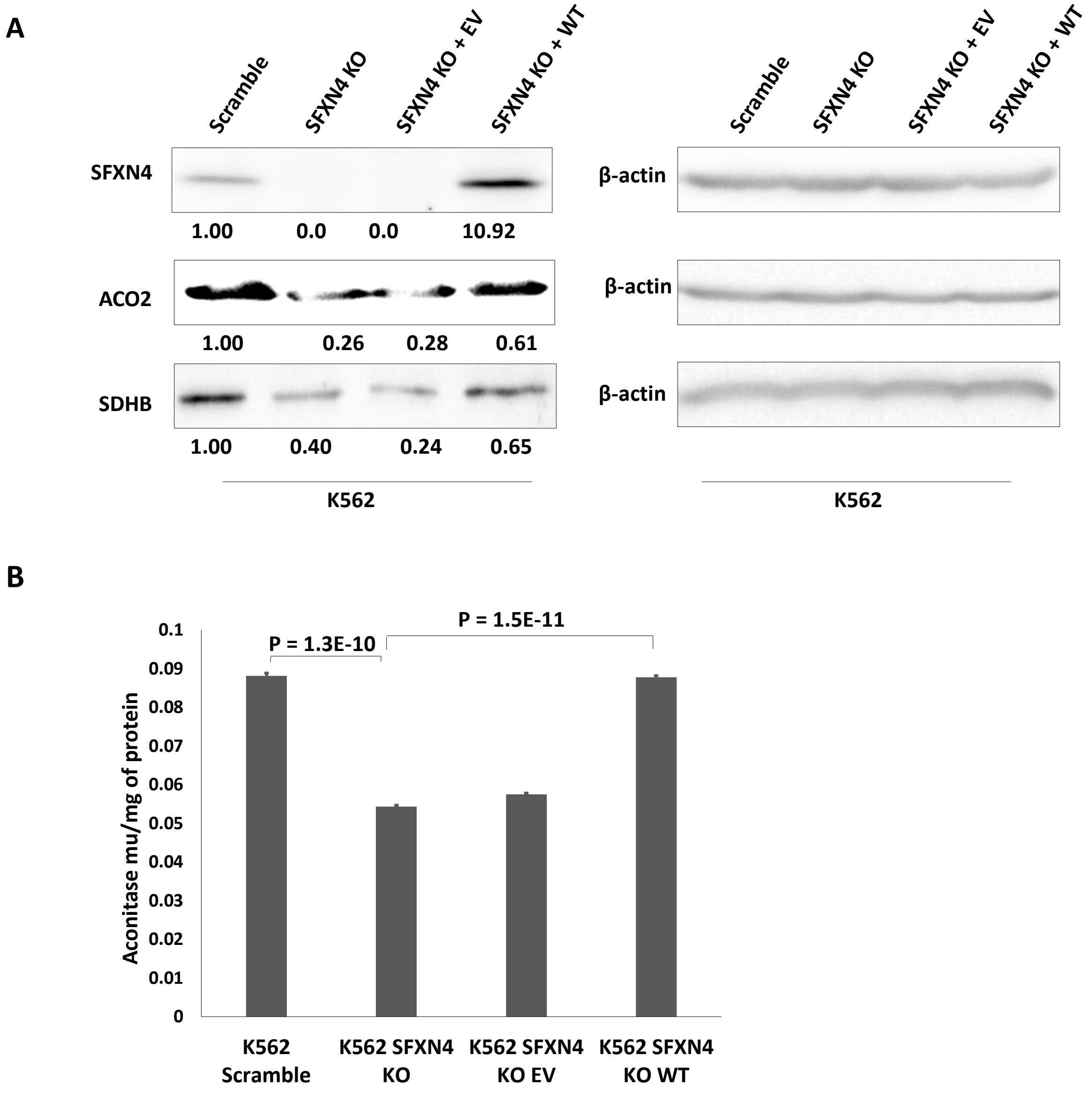

Supplementary Figure 6: Transient reintroduction of SFXN4 in SFXN4 KO cells restores the activity and stability of Fe-S cluster proteins. A) Immunoblot showing the levels of SFXN4, ACO2, and SDHB in K562 cells (scramble, SFXN4 KO, SFXN4 KO cells overexpressing empty vector (EV) and SFXN4 KO cells overexpressing wild type (WT) SFXN4). B) Cytosolic aconitase activity in scramble, SFXN4 KO, SFXN4 KO EV and SFXN4 KO WT K562 cells.

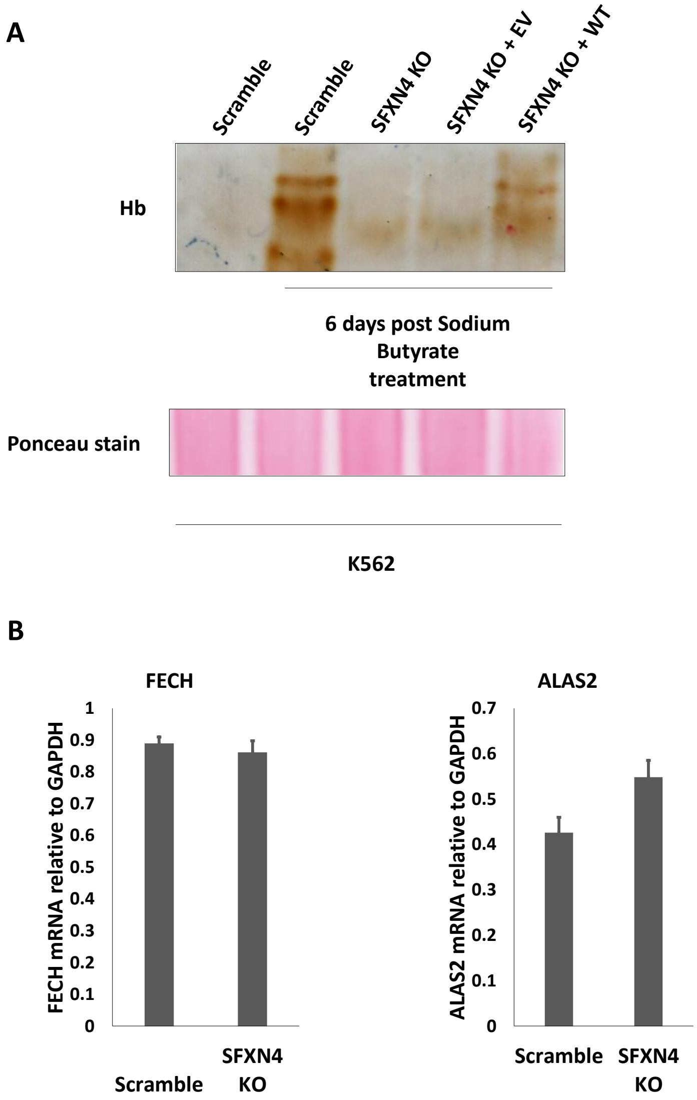

Supplementary Figure 7: Reintroduction of SFXN4 in SFXN4 KO cells restores hemoglobinization and SFXN4 knockout does not decrease mRNA levels of FECH or ALAS2 in K562 cells. A) Hemoglobin in differentiated K562 (scramble, SFXN4 KO, SFXN4 KO EV and SFXN4 KO WT) cell at 6 days post induction of differentiation with sodium butyrate. B) mRNA was quantified by qRT-PCR and normalized to GAPDH. Means and standard deviations of triplicate determinations.
